# Fatal pulmonary tumour thrombotic microangiopathy in patient with ovarian adenocarcinoma: review and a case report

**DOI:** 10.1186/s12872-021-02434-3

**Published:** 2022-01-05

**Authors:** Gintare Neverauskaite-Piliponiene, Kristijonas Cesas, Darius Pranys, Skaidrius Miliauskas, Lina Padervinskiene, Jolanta Laukaitiene, Giedre Baksyte, Gintare Sakalyte, Egle Ereminiene

**Affiliations:** 1 Department of Cardiology, Medical Academy, Lithuanian University of Health Sciences, 44307 Kaunas, Lithuania; 2 Institute of Cardiology, Lithuanian University of Health Sciences, 44307 Kaunas, Lithuania; 3 Department of Pathology, Medical Academy, Lithuanian University of Health Sciences, 44307 Kaunas, Lithuania; 4 Department of Pulmonology, Medical Academy, Lithuanian University of Health Sciences, 44307 Kaunas, Lithuania; 5 Department of Radiology, Medical Academy, Lithuanian University of Health Sciences, 44307 Kaunas, Lithuania

**Keywords:** Pulmonary tumour thrombotic microangiopathy, Ovary adenocarcinoma, Right heart failure

## Abstract

**Background:**

Pulmonary tumour thrombotic microangiopathy (PTTM) is a fatal disease in which tumour cells embolize to the pulmonary vasculature leading to pulmonary hypertension and right heart failure. Early diagnosis is essential for timely treatment which can reduce intimal pulmonary vascular proliferation and prolong survival, improve the symptoms. Due to rare occurrences and no clear diagnostic guidelines the disorder usually is found post-mortem. We present a review of this rare disease and a case of post-mortem diagnosed pulmonary tumour thrombotic microangiopathy in a young female.

**Case presentation:**

51 years old woman presented with progressively worsening dyspnea, right ventricular failure signs and symptoms. Computerized tomography denied pulmonary embolism. 2D transthoracic echocardiography demonstrated right ventricle dilatation and dysfunction, severely increased systolic pulmonary pressure. Right heart catheterization revealed pre-capillary pulmonary hypertension with mean pulmonary artery pressure of 78 mmHg, pulmonary wedge pressure of 15 mmHg, reduced cardiac output to 1.78 L/min with a calculated pulmonary vascular resistance of 35 Wood units, and extremely low oxygen saturation (26%) in pulmonary artery. Because of worsening ascites, pelvic magnetic resonance imaging was performed, tumours in both ovaries were diagnosed. Due to the high operative risk, detailed tumour diagnosis surgically was not established. The patient developed progressive cardiorespiratory failure, unresponsive to optimal heart failure drug treatment. A postmortem morphology analyses revealed tumorous masses in pre-capillary lung vessels, right ventricle hypertrophy, ovary adenocarcinoma.

**Conclusions:**

An early diagnosis of PTTM is essential. Most cases are lethal due to respiratory failure progressing rapidly. Patients with a history of malignancy, symptoms and signs implying of PH should be considered of having PTTM. If detected early enough, combination of chemotherapy with specific PH therapy is believed to be beneficial in reducing intimal proliferation and prolonging survival, along with improving the symptoms.

## Background

Pulmonary tumour thrombotic microangiopathy (PTTM) can be described as pulmonary tumour embolism. It is a rare, fatal and underdiagnosed paraneoplastic syndrome. Tumour cells travel through pulmonary arteries and obstruct small pulmonary vessels, causing substantial reduction of pulmonary circulation [1, 2]. PTTM causes pulmonary hypertension (PH), respiratory failure leading to death due to the right heart failure [3]. Pathologically, major role is played by factors expressed by tumour cells—vascular endothelial growth factor (VEGF), tissue factor (TF) and platelet-derived growth factor (PDGF) [4, 5]. Most recent systematic review consisting of seventy-nine publications with 160 patients, presents PTTM incidence around 1.4–3.3% in patients with carcinoma [6]. The incidence can be higher depending on the primary location of the tumour causing PTTM. Most common primary tumour—gastric (59%), less common—breast cancer (10%), lung cancer (6.3%), urothelial carcinoma (3.8%), ovarian carcinoma (2.5%). Unfortunately, due to the intricacy of the symptoms, no diagnostic guidelines and a rapid lethal progression of the right heart failure most diagnosis (around 79%) are made post-mortem and only a few (around 9.4%) ante-mortem. The frequency of reported PTTM cases has been increasing recently putting a highlight on the subject. To give input into we believe it is important to share our experience. We present a case of post-mortem diagnosed PTTM in young female with a rapidly progressing PH and right heart failure with primary ovarian tumour.

## Case presentation

51-year-old woman presented with progressively worsening dyspnea for 6 months.

Prior to hospitalisation a viral infection was diagnosed and patient was on antiviral treatment at home. Despite the treatment, dyspnea progressed. Pulmonary embolism (PE) was suspected, and patient was hospitalised to the local hospital. PE was ruled out by computerized tomography pulmonary angiography (CTPA). The abdominal and pelvic computerized tomography were done due to the ascites and revealed bilateral ovarian masses. The right heart failure of an unknown cause was suspected, and the patient was transferred to University hospital’s cardiac intensive care unit.

**Fig. 1.**
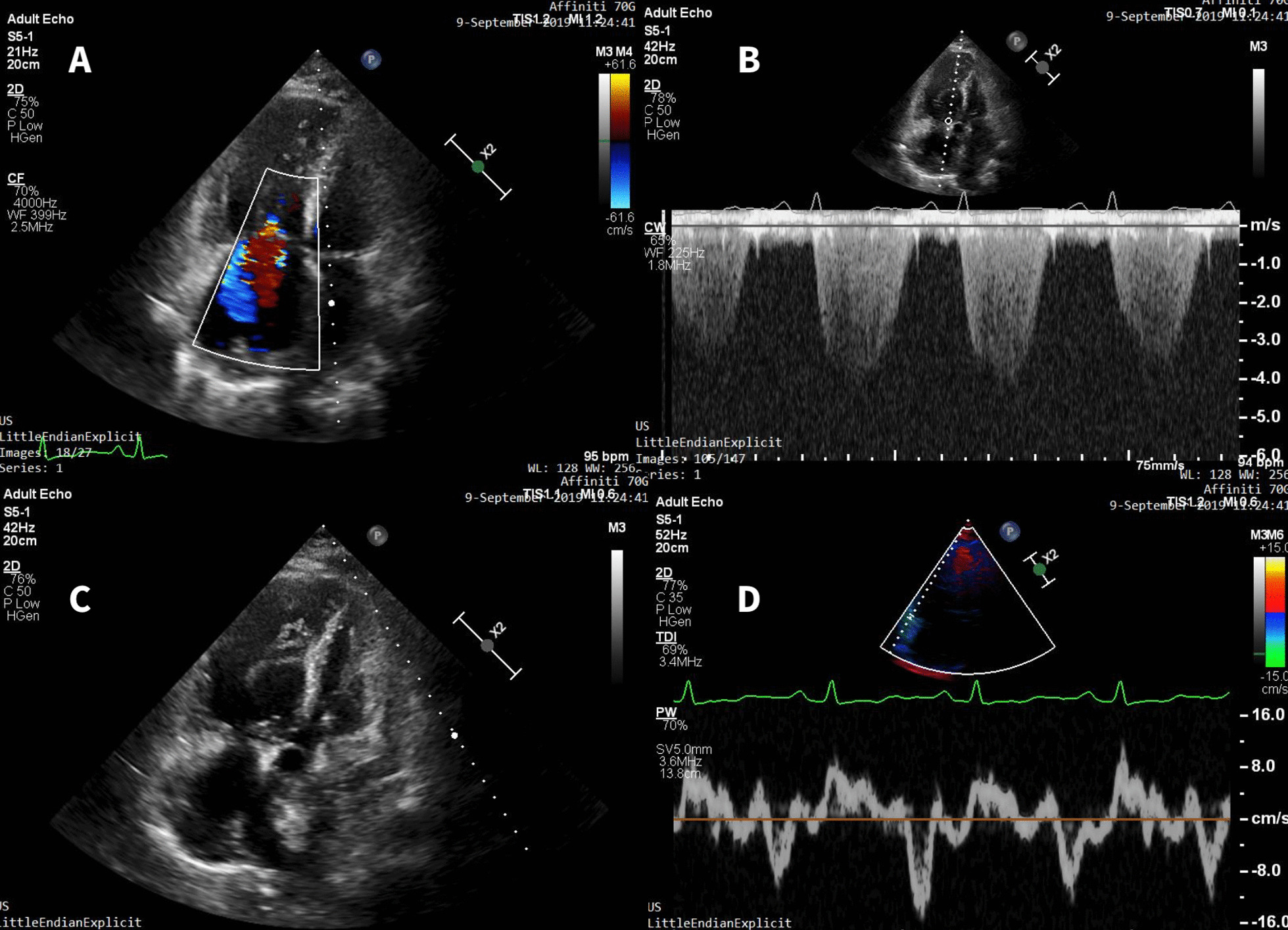
2D Doppler echocardiography—four chambers apical view A, B, C, D. Revealing tricuspid regurgitation, maximal tricuspid regurgitation velocity (TR Vmax)—4.1 m/s indicating pulmonary hypertension, right heart enlargement and reduced RV free wall longitudinal function (S’—8.0 cm/s)

**Fig. 2:**
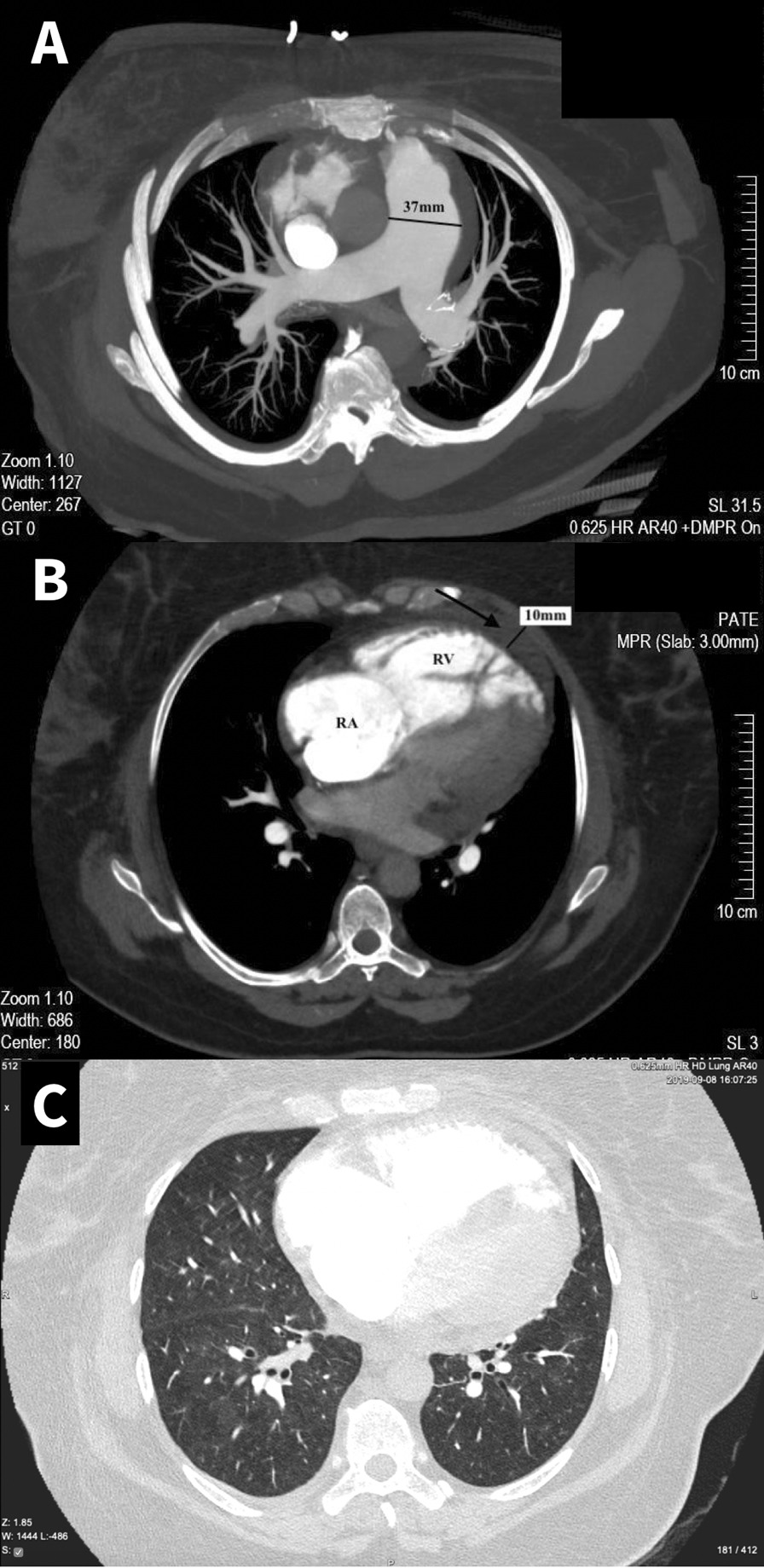
Reconstruction of computerized tomography pulmonary angiography (CTPA). Axial CT angiography scans: **A** the dilatation of the pulmonary artery trunk and both main pulmonary arteries with no filling defects within pulmonary vasculature. B The dilatation of right ventricle (RV), atrium (RA) and pericardial effusion (black arrow). **C** multiple perivascular areas of hyperattenuation in keeping with PH. Note the increased segmental artery-to-bronchus ratio up to 2,5:1

On presentation the patient was hypotensive (blood pressure 100/70 mmHg) and hypoxemic (SpO2 of 88% on room air). Chest radiograph revealed cardiomegaly and no infiltration or pleural effusion. Laboratory results showed reduced kidney function (creatinine 144 μmol/L), electrolyte balance (potassium 3.1 mmol/l), elevated Hb (173 g/l), thrombocytopenia (67 × 10^9/l), severely elevated NT-proBNP levels (6799 ng/l). 2D transthoracic echocardiography demonstrated dilation of the right atrium and ventricle, dysfunction of the right ventricle (RV), severe tricuspid regurgitation with calculated systolic pulmonary artery pressure of 82 mmHg, small pericardial effusion (Fig. 1). CTPA did not show any signs of PE within pulmonary vasculature, but pulmonary hypertension was strongly suggested: central pulmonary artery dilatation (37 mm), right ventricular and atrial enlargement, dilated bronchial arteries (segmental artery-to-bronchus ratio up to 2.5:1), pericardial effusion (10 mm) were found (Fig. 2).

Right heart catheterization revealed extremely low saturation in pulmonary artery of 26% and severe pre-capillary pulmonary hypertension with mean pulmonary artery pressure (mPAP) of 78 mmHg. Pulmonary capillary wedge pressure was 15 mmHg and a cardiac output (CO) of 1.78 L/min with a calculated pulmonary vascular resistance of 35 Wood units were established.

Magnetic resonance imaging of the abdomen revealed tumors in both ovaries. These results were discussed with gynecologist in the multidisciplinary team meeting. Surgical procedure was proposed for the proper diagnosis, however, it was not possible due to the worsening of right heart function. The main ovarian cancer antigen 125 (Ca 125) was elevated at 704.3 kU/l (reference range 0–35 kU/l), other cancer antigens (Ca 19–9, Ca 27,29) were within normal ranges. Transvaginal ultrasound confirmed dermoid cyst in right ovary and tumor of unknown origin in the left one.

**Fig. 3:**
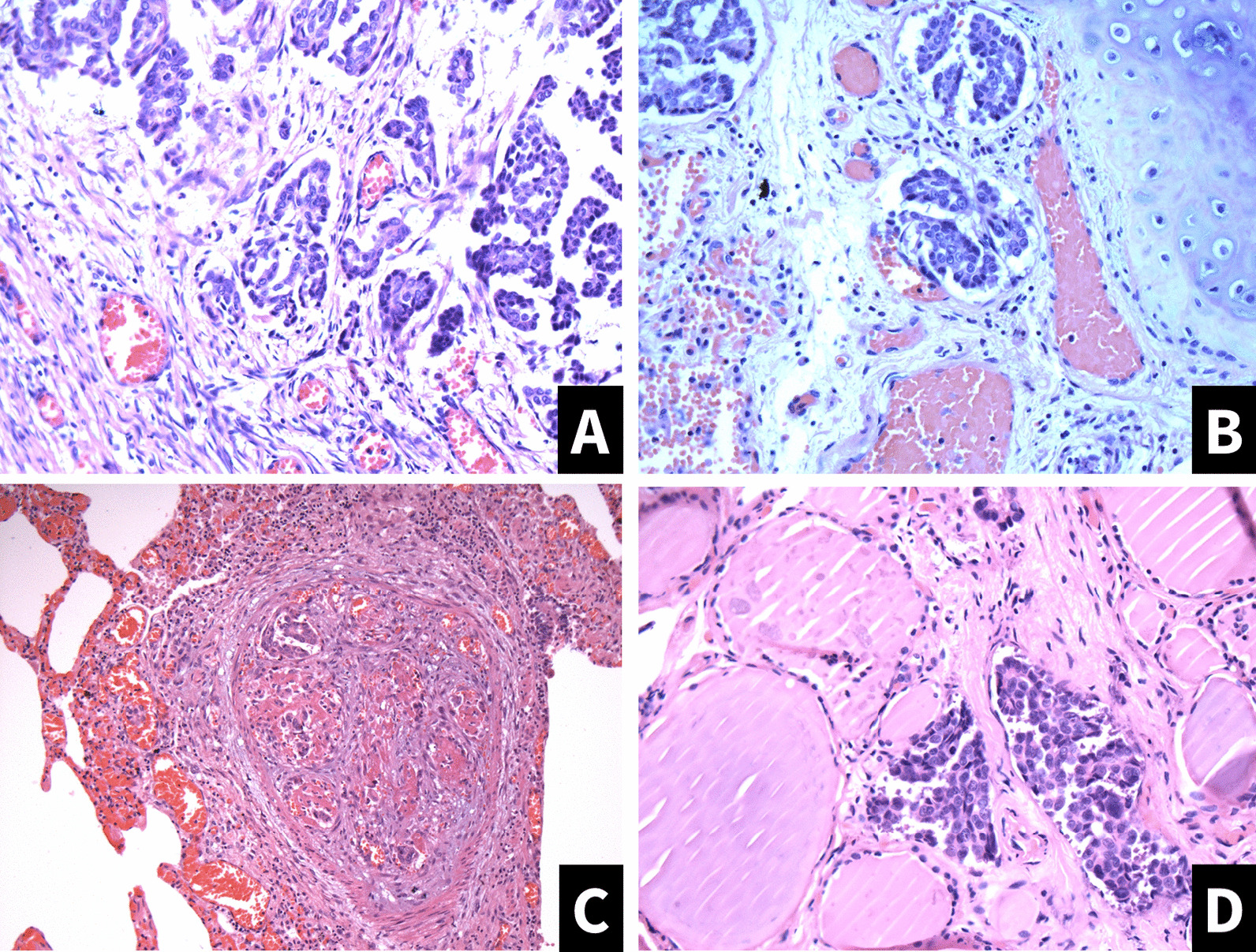
Postmortem histological evaluation results (hematoxylin and eosin staining). **A** Microscopic findings in left ovary with low-grade serous carcinoma—moderately atypical cells with round chromatic nuclei and moderate amount of pale eosinophilic cytoplasm are observed; tumor cells form solid pattern and some primitive papillary structures. **B**, **C** Microscopic findings in pulmonary tissue—multiple veins and lymphatic vessels, some of these vascular lumens are obliterated by tumorous thrombi consisting of ovarian tumor`s structures (picture B); dilated vein with tumorous thrombus which is partially recanalized (picture C). **D** Microscopic findings in thyroid gland tissue—similar to those observed in pulmonary tissue

Patient was treated with intravenous diuretics, digoxin, low-molecular-weight heparin, ivabradine. During the treatment SpO2 was approximately 84–90% with additional oxygen through the nasal cannula, arterial blood gas examination revealed low partial oxygen pressure of 55 mmHg and low partial carbon dioxide pressure—23.2 mmHg, SpO2—90.4%, and respiratory alkalosis with pH level of 7.52. Connective tissue, infective, lung diseases were excluded. Multidisciplinary team meeting was scheduled to discuss specific pulmonary hypertension treatment, but on the 17^th^ day of admission the patient became acutely hypoxic, and despite treatment, including mechanical ventilation, circulatory support, the patient died. A postmortem examination was performed and revealed tumorous masses in lung vessels formed by ovarian tumor structures, right ventricle and septum hypertrophy, low-grade serous carcinoma in the left ovary as well as changes in thyroid gland tissue—similar to those observed in pulmonary tissue (Fig. 3). A post-mortem diagnosis of PTTM was made.

## Discussion and conclusions

First described in 1990 by von Herbay et al. [1], PTTM is known as a rare complication of cancer. It is called as “paraneoplastic syndrome” by some authors [2, 3]. As PTTM progresses, the proliferation of pulmonary arterial endothelium and hypercoagulation occurs. This leads to narrowing and occluding of small pulmonary vessels until pulmonary hypertension, dyspnea, respiratory failure develop [1, 6]. A major part here is played by vascular endothelial growth factor (VEGF) [4]. It has been previously reported that VEGF is being expressed by tumour cells in PTTM cases, as well as tissue factor (TF) and platelet-derived growth factor (PDGF) [5]. The expression of TF and PDGF can lead to hypercoagulation state causing disseminated intravascular coagulation (DIC) [4, 7]. This mechanism provokes PH by inducing the remodelling of pre-capillary and post-capillary pulmonary vessels [8]. According to the World Health Organization's classification system, PTTM can be placed into Group 5 PH category [9].

A systematic review by Godbole et al. reported a total of 59% PTTM cases with gastric adenocarcinoma as primary malignancy, 10% breast cancer, 6.3% lung cancer, 3.8% urothelial carcinoma and only 2.5% of ovarian carcinoma [6]. The complexity of diagnostics and the rapid progress of PTTM usually lead to a post-mortem diagnosis. Now that autopsy is not performed in every patient, PTTM can go underdiagnosed [6].

Ante-mortem diagnosis is extremely challenging due to the rapid development of lethal pulmonary hypertension. High level of clinical suspicion is required during PTTM cases. Clinical manifestation is not specific and can be mistaken for other diseases, such as chronic thromboembolic pulmonary hypertension (CTPH). The most common symptom reported is shortness of breath that usually rapidly progresses from exertional to a resting dyspnea [10]. This can be caused by PH, hypoxemia or as a result of obstruction [11]. Cough can also be seen, usually dry and with a tendency to progress [12, 13]. Other symptoms may be related to the primary tumour, such as abdominal pain for gastric, or a common symptom—weight loss. As PTTM patient’s clinical status can progress very rapidly and lead to worse prognosis, it is important to recognize the possibility of PTTM and take further actions.

Laboratory results often show anaemia, elevated D-dimer concentration. As a result of DIC, thrombocytopenia can also occur. Laboratory findings are not specific for PTTM but should be considered along with other findings [6].

Chest radiograph is not sensitive or specific when diagnosing PTTM—even if it’s unremarkable further investigations should be taken [14]. Chest computerized tomography (CT) scan can be helpful, but specificity is low [6]. Godbole et al. describes CT features which can be seen in severe tumor embolization and vascular remodelling: septal thickening, tree-in-bud sign, small semi solid nodules, and non-solid central lobular ground glass opacification [15]. 2D echocardiography is essential to suspect PH and evaluate changes of RV geometry and function. However, histological, invasive tests are superior compared to imaging tests or laboratory tests when suspecting PTTM. Right heart catheterization confirms the diagnosis of precapillary hypertension, and pulmonary artery catheterization with wedged pulmonary arterial blood cell sampling have been reported as a successful method for confirming the diagnosis with sensitivity of 80–88% and specificity of 82–94% [6, 16, 17]. Lung histology findings usually reveal tumour cells and fibrocellular intimal hyperplasia. These imaging tools are important as they can lead to a suspicion of PTTM, especially if any information of possible malignancy is present.

To sum up, PTTM clinical symptoms mimic many other possible syndromes and diseases, for example CTPH. In order to differentiate the cause of dyspnea (the most common symptoms) the specific diagnostic approach should start with imaging tests. 2D echocardiography would reveal PH and right heart failure. CTA would deny the pulmonary embolism, possibly reveal septal thickening, nodules and mediastinal/hilar lymphadenopathy—features that has high sensitivity for PTTM diagnosis [6]. Next step should be invasive tests. In order to, confirm PH, pulmonary artery catheterization should be performed as well as wedged pulmonary arterial blood cell sampling. The catherization will confirm the diagnosis of PH and cell sampling will show tumour cells. According to Godbole, as well as arterial blood cell sampling, lung biopsy also has a high specificity and sensitivity. However, it should only be performed if patient status allows it, since PTTM patient status usually deteriorates very quickly.

Although there is no standard management protocol to follow, the aim of the treatment should be focused on elimination of malignancy and improving the pulmonary pressure, cardiac output. Few reports have shown that imatinib helped to improve survival. Since imatinib is a PDGF receptor inhibitor, it may reduce the vascular remodelling that causes PH [6]. Yochikawa et al. reports a patient with metastatic breast cancer to have an improvement of PH after one month of imatinib administration. Although the patient died few months after discharging due to extremely aggressive primary malignancy, imatinib is believed to have alleviated the prognosis and symptoms [18]. In addition to imatinib, chemotherapy focusing the primary cause of PTTM is mandatory. In one primary gastric cancer case imatinib and a combinational chemotherapy (TS-1) was used and significant PH improvement was reported after 16 days [19]. Other author reports a treatment with combination chemotherapy S-1 and cisplatin, which improved patient’s symptoms and showed improvement in CT findings [10]. Advanced PH treatment also should be considered in PTTM cases. It prolongs survival, by controlling the PH progression [6]. However, all cases reporting any PH treatment with phosphodiesterase inhibitors, endothelin-receptor antagonist or prostacyclin analogue, combines treatment with anti-neoplastic drugs, mainly—imatinib [19, 20]. Treatment in such cases as ours should focus on location specific chemotherapy and PH treatment in combination with imatinib. This allows to focus on eliminating the primary tumors as well as reduces symptoms of PH. Cases report a positive effect of this treatment: prolonged survival, reduced symptoms. However, in many cases PTTM progresses slower but remains lethal for the patient.

There are no strict guidelines how to proceed with a patient. It is important that the treatment strategy must be planned by a multidisciplinary team, after considering patient’s medical history, primary tumor site and current clinical status. Further analysis and research is required to establish the most successful direction in PTTM treatment.

In our case, PTTM was caused by ovarian adenocarcinoma, a rare primary malignancy for PTTM. Young patients age, the rareness of the disease led us only to post-mortem diagnosis. Severe precapillary pulmonary hypertension was diagnosed with rapidly progressing right ventricular failure and hypoxemia. All the investigations ruled out other possible aetiologies of PH. Specific invasive investigations such as arterial blood cell sampling, lung biopsy were not performed. Transvaginal ultrasound confirmed tumours in both ovaries together with elevated levels of the main ovarian cancer antigen 125. Still, surgery was impossible because of patient’s poor performance status.

The symptomatic treatment was ineffective, chemotherapy and specific pulmonary hypertension treatment was not started in this current case, due to the patient’s rapid deterioration and death. Post-mortem examination revealed tumorous masses formed by ovarian tumor masses obliterating lung vessels.

The clinical course of this case provides us an important lesson. According to systematic review by Godbole et al. ovarian tumors are responsible for only 2.5% of PTTM cases, which makes our case exceptionally rare and as many similar cases was diagnosed post-mortem. We believe that this publication will contribute to PTTM patients in the future in order to suspect, diagnose and manage the disease early. It is important to suspect the PTTM in any PH case combined with oncology. Early diagnosis is essential due to rapidly progressive symptoms, for which specific treatment could be applied. Improvement in symptoms and prolonged survival could be reached with specific treatment.

In conclusion, an early diagnosis of PTTM is essential. Respiratory failure progresses rapidly, caused by PH and right sided heart failure, which in most cases is lethal. Patients with a history of malignancy and symptoms implying of PH should be considered of having PTTM and specific diagnostic tools, including noninvasive as well as pulmonary artery catheterization as well as wedged pulmonary arterial blood cell sampling should be performed to confirm this diagnosis. If detected early enough, combination of chemotherapy with specific PH therapy is believed to be beneficial in reducing intimal proliferation and prolonging survival, along with improving the symptoms. Nevertheless, PTTM requires more clinical research for timely diagnosis and treatment.

## Data Availability

All relevant data supporting the conclusions of this article are included within the article.

## Abbreviations

PTTM: Pulmonary tumour thrombotic microangiopathy
PH: Pulmonary hypertension
VEGF: Vascular endothelial growth factor
TF: Tissue factor
PDGF: Platelet-derived growth factor
PE: Pulmonary embolism
CTPA: Computerized tomography pulmonary angiography
RV: Right ventricle
CO: Cardiac output
Ca: Cancer antigen
DIC: Disseminated intravascular coagulation
CT: Computerized tomography
FDG: 18-Fluoro-deoxyglucose
PET: Positron emission tomography
